# Virtual friendly visitor program: combatting loneliness in community dwelling older adults

**DOI:** 10.3389/fpubh.2024.1440465

**Published:** 2024-12-11

**Authors:** Barbara A. Gordon, Chelsea B. Miceli, Pamela A. Yankeelov, Samantha G. Cotton, Anna C. Faul

**Affiliations:** ^1^Trager Institute, University of Louisville, Louisville, KY, United States; ^2^Kent School of Social Work and Family Science, University of Louisville, Louisville, KY, United States; ^3^Department of Family and Geriatric Medicine, School of Medicine, University of Louisville, Louisville, KY, United States

**Keywords:** loneliness, social networks, virtual friendly visitors, older adults, social work student interns

## Abstract

**Introduction:**

Loneliness is a critical public health issue affecting older adults, with significant impacts on their mental and physical health, including increased risks of depression, cognitive decline, and higher mortality rates, necessitating distinct approaches for each condition given their unique implications and the exacerbation of these issues during the COVID-19 pandemic. We examine the implementation and outcomes of a Friendly Visitor Program (FVP) designed to mitigate loneliness among older adults. The program involved social work student interns providing virtual visits to older adults using computers and tablets, with the goal of enhancing social interaction and support.

**Methods:**

The study utilized a qualitative narrative design for process evaluation and a longitudinal non-experimental, prospective research design for outcome evaluation, employing a three-level cross-classified longitudinal growth model to assess changes in loneliness among VFVP participants while also testing potential predictors of these changes.

**Results:**

Findings indicated that the program was associated with reduced loneliness over time. Younger and White participants performed better in the program than older participants from other races and ethnicity. Satisfaction with visits and willingness to recommend the program were significant predictors of reduced loneliness. Unexpectedly, greater comfort with technology correlated with increased loneliness, suggesting overreliance on digital interactions may not substitute for in-person contact. Furthermore, improved social networks was associated with reduced loneliness, highlighting the importance of strong social networks.

**Discussion:**

The study underscores the potential of friendly visitor interventions in addressing the challenges of lonely older adults and provides insights for optimizing such programs in the future.

## Introduction

Loneliness in older adults is a complex and multidimensional concept that extends beyond a subjective feeling of social isolation. It encompasses social, emotional, and existential dimensions, each contributing uniquely to the experience of loneliness. Social loneliness arises from a lack of engagement with a broader network of meaningful social connections, which is often exacerbated in older adults by reduced participation in social activities and retirement (1). Emotional loneliness, on the other hand, is defined by the absence of close emotional bonds, such as those formed with a partner or confidant. This form of loneliness becomes particularly pronounced following the loss of a spouse or significant others, as it disrupts the intimacy and support these relationships provide (2). Existential loneliness reflects a deeper sense of isolation. It emerges from an awareness of one’s separateness and mortality, often accompanied by feelings of emptiness, alienation, and a lack of purpose. This dimension is especially relevant among older adults facing declining health, end-of-life considerations, or reflections on the meaning of their lives (3, 4). To effectively assess and mitigate loneliness, it is crucial to acknowledge its multifaceted nature and tailor interventions to the individual’s specific experiences (4, 5). This paper describes a Virtual Friendly Visitor Program (VFVP), its impact, challenges and role in addressing older adults’ loneliness, specifically the social and emotional dimensions of loneliness. Loneliness is increasingly recognized as a critical public health challenge affecting older adults, with profound impacts on their mental and physical health, due to the emotional strain from not having meaningful connections. Loneliness is intricately linked to a range of negative health outcomes in older adults, including depression, anxiety, cognitive decline, and high mortality rates (6–8).

With the onset of COVID-19 and the subsequent social distancing measures introduced to curtail the virus’s spread further isolated many older adults from their communities, family members, and support networks (9). The pandemic necessitated widespread social distancing measures, intended to curb the virus’s spread but also resulting in profound social and psychological impacts (10). Restrictions on gatherings, closure of community centers, and the general hesitancy around in-person interactions meant that many typical venues for social engagement were suddenly inaccessible. This situation is particularly alarming as it not only affects the quality of life but also the longevity of the older population (7).

To address these challenges, Friendly Visitor Programs (FVP) have long been used to combat loneliness among older adults by offering social interaction and social networking through regular visits from volunteers (11, 12). Historically supported by community-based services and funded through the Older Americans Act, friendly visiting is a well-established intervention. The Older Americans Act (OAA) of 1965 states the Administration on Community Living will provide grants to states to support a variety of supportive services, including those that “promote or support social connectedness and reduce negative health effects associated with social isolation” (13). As a result of this provision services such as telephone reassurance and friendly visiting were developed as part of the Area Agency on Aging Network and have been provided as part of the home and community-based services alignment for many decades.

Social networking programs like FVPs are associated with combatting isolation by providing various types of assistance that alleviate feelings of loneliness. FVPs can fill the gap between social network needs and actual social connections, thus reducing loneliness. Social networking programs that emphasize building and maintain friendships has the potential to be associated with reduced subjective feelings of loneliness (14). People often gravitate toward friends who share similar interests, values, and backgrounds, as these commonalities foster a sense of understanding and connection. This tendency is rooted in the comfort and affirmation found in interacting with others who reflect familiar aspects of themselves (15, 16). Engaging in conversations, participating in social activities, and maintaining interpersonal relationships are crucial for mental agility and emotional health. Without these interactions, older adults are at a higher risk of cognitive decline, including memory loss and reduced problem-solving abilities (17).

The Trager Institute at the University of Louisville in Kentucky, USA has provided services to community-dwelling older adults for the past 10 years as part of our Health Resources Services Administration (HRSA) funded Geriatric Workforce Enhancement Program (GWEP). The program focuses on improving health care for older adults and maximizing patient and family engagement by training the future healthcare workforce to provide age-friendly services to older adults and to develop programs and systems that improve health outcomes for older adults. Every year the Institute trains over 700 interprofessional healthcare learners from medicine, nursing, dentistry, pharmacy, and social work to develop skills in working with older adults, offering an intensive, two-semester practicum for social work interns (18–22).

COVID-19 highlighted the need to develop virtual social networking programs where physical contact would be eliminated, allowing isolated older adults the advantage of creating friendships and social networkst via tablets and video technology. Research has shown that better access to technology, and better proficiency in using technology can promote connectivity and a sense of belonging. Social media and video chat platforms offer unique opportunities to make new friends and share information about life events with friends and family. Internet use has been associated with decreased loneliness as it is seen as a vehicle for maintaining social contact (23).

The exacerbation of social isolation and loneliness among older adults during the COVID-19 pandemic highlighted a dual challenge for the Institute: the immediate need to address the acute impacts of the pandemic on loneliness and the broader requirement to develop sustainable strategies to combat loneliness and isolation in the long term. Utilizing COVID-Cares Act funds, our GWEP adapted an in-home friendly visitors program to a Virtual Friendly Visitor Program (VFVP) to meet the needs of our patients and community members across Kentucky by purchasing easy to use tablets that could be used by participants to engage in virtual friendly visits. Building on the core principles of Friendly Visitor programs, we designed our initiative to reduce loneliness by leveraging a network of Bachelor Level Social Work and Foundational Master Level of Social Work students from our internship program to become virtual visitors to isolated older adults. Experience has taught us that personal experiences with older adults enrich learners’ professional development, making them more empathetic, skilled, and effective in delivering high-quality care to this population. The social work interns were in the unique position where they could engage virtually with older adults as visitors in the VFVP to help them develop the skills they will need in their future careers.

This paper examines the development and implementation of the VFVP, its effects on participants, and the challenges encountered, emphasizing the unique impact of COVID-19 on older adults’ loneliness and the critical role of virtual social networks during and after the pandemic. The study’s uniqueness lies in the combination of its approach, methodologies, and comprehensive analysis, contributing significantly to the existing literature on loneliness mitigation and the implementation of friendly visitor programs among older adult populations. Our FVP program leverages social work student interns to provide virtual visits to older adults using digital devices such as computers while fosters intergenerational interaction. By further demonstrating the effectiveness of friendly visitor interventions and identifying key predictors of success, our research provides valuable evidence for developing and optimizing similar programs.

## Conceptual framework

The Model of Depression and Loneliness (MODEL) (11, 24, 25) provides a theoretical and structured approach to understanding how various factors contribute to depression and loneliness, particularly in older adults. The model is rooted in a cognitive-behavioral theory that conceptualizes behaviors as resulting from an interaction of cognitive processes and environmental events (26). It emphasizes the interplay between individual characteristics, environmental factors, and social network systems.

Individual characteristics that contribute to depression and loneliness are psychological factors like mental health status, negative thought patterns, physical health constraints that can influence an individual’s ability to engage socially and maintain relationships, and demographics like age, gender and socioeconomic status (27, 28). Environmental factors that contribute to depression and loneliness are the living arrangements of older adults that may limit the ability to have social interactions and can increase feelings of loneliness (29). Lack of access to community resources can also exacerbate feelings of isolation. The lack of social networks in the form of quality relationships with family and friends that can provide emotional support, companionship, and a sense of belonging can lead to feelings of loneliness (30, 31). In addition, lack of access to formal support services, e.g., counseling and support groups and visiting programs may add additional layers of stress (32).

FVPs can provide regular interaction with frequent visits between volunteers and older adults. These visits provide consistent social contact, reduce feelings of loneliness and improve emotional well-being, building a reliable support network. Visitors can offer empathy, companionship, and a listening ear, all important for older adults who may lack strong social ties. Engaging in meaningful conversations and activities during visits can improve mood and reduce depressive symptoms. Visitors can encourage positive thinking and coping strategies. It also provides a sense of purpose to older adults by providing something they can look forward to. Finally, FVPs can help connect individuals with community resources as well as in-home socializing. By enhancing social networks, FVPs can improve psychological well-being, and mitigate environmental barriers, thereby reducing loneliness among older adults (11, 33).

## Methods and procedures

### Intervention

We followed the Template for Intervention Description and Replication (TIDieR) checklist and guide to describe the key elements of our VFVP (34). TIDieR is a 12-item checklist that was developed to guide the reporting of interventions with the goal of maximizing reproducibility (35). The *main purpose* of our VFVP was to reach lonely older adults living in the community, with the *intended outcome* of being associated with reduced loneliness. Our VFVP used a *befriending program* to provide a meaningful and personalized response to the loneliness that is individualized to the needs of the older adult participant. According the Cacioppo Evolutionary Theory of Loneliness (ETL), loneliness serves as a biological warning signal that alerts individuals to potential damage to their social connections and motivates them to repair or replace these relationships. Loneliness therefore could increase the motivation to attend to and approach social stimuli to repair or replace deficient social relationships. Our VFVP program provided regular and meaningful social interactions, emotional support, practical assistance and a sense of belonging, thereby helping individuals to rebuild and maintain social connections and thus reducing feelings of loneliness (36, 37).

The program *procedurally targeted*: (1) the formation of new and meaningful relationships with volunteers, (2) that would facilitate the provision of informal support and (3) would provide mediated formal support where needed (32). There is significant conceptual and empirical literature available that delineates the mechanism and benefits of social relationships and social networks on an individual’s mental health and well-being (38–40). Socially isolated older adults are more likely to experience positive results from a program if they can form *new and meaningful relationships with visitors*. Therefore, visitors in our VFVP were trained in making genuine human connections with the participants they visited to ensure that the key elements of the newly formed participant-visitor relationship were reciprocity, reliability, and authenticity. The *provision of informal support to the participant* by the visitor was based on the ability of the visitor to develop a friendship with the older adult participant that included support in the form of socialization, personal assistance and advice. As visitors and isolated participants met regularly, the assumption was that new and trusting relationships would begin to form, and the participant would begin to experience the benefits of informal support. Activities that were promoted in this befriending program were engagement in everyday life activities through dialogue, discussing mutual interests, sharing life stories, providing casual advice, being an active listener and providing an emotional connection. Providing additional *social networks* was an additional focus of our VFVP, as visitors were trained in having knowledge of local resources and support services and introducing the participants to these local support networks. The Institute acted as the host organization who behind the scenes provided guidance to visitors on how to connect isolated participants to these valuable resources (32).

*Different entities* were involved in the VFVP program. The Institute acted as the host organization, bringing together a vast array of knowledge surrounding the care of older adults as well as in-depth understanding of formal support services in different communities that could support lonely and isolated older adults. Staff at the Institute with advanced degrees in counseling and social work, acted as program supervisors for the VFVP program. The visitors were student interns from undergraduate and graduate social work programs completing internship placements at the Institute. Before engaging with any of the VFVP participants, the interns were trained in a 4-h training session on how to administer the program. Each training session was conducted through in-person, virtual, and hybrid modalities. The curriculum was designed to cover key aspects of the program, focusing on training the student interns to build connections with participants and understanding the logistics of program implementation. Matching between visitors and participants were done based on interests and preferences of the participants documented in an intake survey. Visitors were required to contact their assigned participant within 48 h of participant enrollment. They received weekly supervision from well-trained supervisors to support meaningful engagement with the participants.

The VFVP was delivered in *one-on-one weekly individualized virtual sessions* between visitors and older adult participants. The visits were done virtually to allow for a wider reach and easy access to isolated older adults. There was no time limit established for the intervention with visits continuing until the participant declined further participation. When interns ended their internships at the Institute, new visitors were assigned when participants requested a continuation of the program. The locations for program interactions vary between student intern visitors and participants. Typical settings for visitors included home offices, dorm rooms, libraries, or secured offices. Student interns were trained to conduct sessions from confidential locations, ensuring that conversations with participants remained private. This included wearing headphones if necessary. Participants had the flexibility of engagement in the VFVP regardless of their location. Participants typically received virtual visits within their homes. However, some participants took calls from their vehicles or chose to visit local establishments, such as public restaurants or libraries, to access better Wi-Fi connectivity for the visits. Even though the VFVP was planned to include weekly visits, it was at times necessary to *tailor* the program to the individual needs of both participants and visitors. When visitors were on university breaks, it sometimes delayed the weekly visits to accommodate this break. Similarly, when participants experienced significant life stressors, they sometimes requested either more visits or fewer visits, depending on the circumstances.

The *technology materials* utilized in our VFVP consisted of smartphones, computers and tablets for those who did not have either a smartphone or computer. We used FaceTime and Zoom for our virtual platform. These two platforms were chosen because they were easily accessible and easy to use. They also could be used beyond the VFVP and facilitate increased social connections. This method has been used in other studies to improve social well-being, reduce loneliness and enhance quality of life (41).

Detailed *training materials* were developed for student interns that included a VFVP Toolkit with outlined policies and procedures, technology training including how to train older adults in the use of technology, specific modules on how to build connections with older adults and use skills such as motivational interviewing techniques to build rapport, training on protocols to manage any emergencies that might occur during and between visits, workflow documents for program implementation, specific training modules on confidentiality protocols including the handling of Health Insurance Portability and Accountability Act (HIPAA)-sensitive information shared by participants, and training on the evaluation component of the program. In addition, monitoring tools were developed and used to provide ongoing support to the visitors. *Marketing materials* were developed and distributed through our wide networking or partnering organizations.

Our VFVP went through a few *modification phases* since 2020. High school students were initially used as visitors for nursing home resident participants with an Area Agency on Aging acting as the host sponsor. These students had to belong to their local chapter of the Health Organization Service Organization (HOSA)-Future Health Professionals, a student-led organization that support leadership development in the global health community through education, collaboration and experience, to participate. Unfortunately, COVID breakouts in the nursing homes, nursing home staff shortages for technology support during the visits and a major flood disaster in Eastern Kentucky affected the viability of this initiative for the AAA sponsor. As a result, the program moved away from nursing homes and was re-envisioned as a new program serving community dwelling older adults who received virtual visits using university student interns. The interns who participated in this program did so as part of their internship, a requirement of their social work degree program. The downside of using student interns was that participant transitions between volunteers were necessitated by the academic term structure, often following the completion of practicum or graduation. To ensure a seamless transition for participants, program policies were established, and a warm hand off was conducted between the existing and new student with the older adult. We have found this practice provided the participant with more comfortability. Furthermore, graduating students were offered the opportunity to maintain engagement with their participants post-graduation, provided they adhere to all program policies and procedures, including documentation.

### Design

The design of the study focused on both a process and outcome evaluation of the VFVP as delivered between December 2021 and March 2024. Data on the pilot period (August 2020–December 2021) where not all visitors were students and where all program training materials for the interns were completed and tested were not included as part of this study. The study was conducted in compliance with our institution’s Internal Review Board (IRB).

For the process evaluation, a qualitative narrative design was used, using visitor completed service records and the visitor evaluations at the conclusion of their commitment. This design allowed for the exploration and understanding of experiences and stories from the visitors to capture the complexity of the visitor experience. This method is many times used where the context and subjective experiences of participants are crucial to understand the topic (42).

For the outcome evaluation a longitudinal non-experimental, prospective research design was utilized. This design allowed the study team to observe and measure the association between loneliness among older adult participants and their participation in the VFVP over an extended period of time. Data were collected at many time points to identify trends of loneliness over time (43, 44). We used a three-level cross classified longitudinal growth model, to examine individual growth differences in loneliness between 2 and 5 measurement occasions while enrolled in the VFVP. We also tested potential predictors that could explain these differences (45).

### Research questions and hypotheses

The main process evaluation questions were: (1) Did the participants receive all the core model components intended by the VFVP? (2) Were the student intern visitors satisfied with the value of the VFV to their learning and what were their perceptions of the program? The process hypotheses were: Process H1: Participants received all the core model components intended by the VFVP; Process H2: Student intern visitors were satisfied with the value of the VFV to their learning; Process H3: Student intern visitors’ perceptions of the program were valuable to understanding their satisfaction.

**Figure 1:**
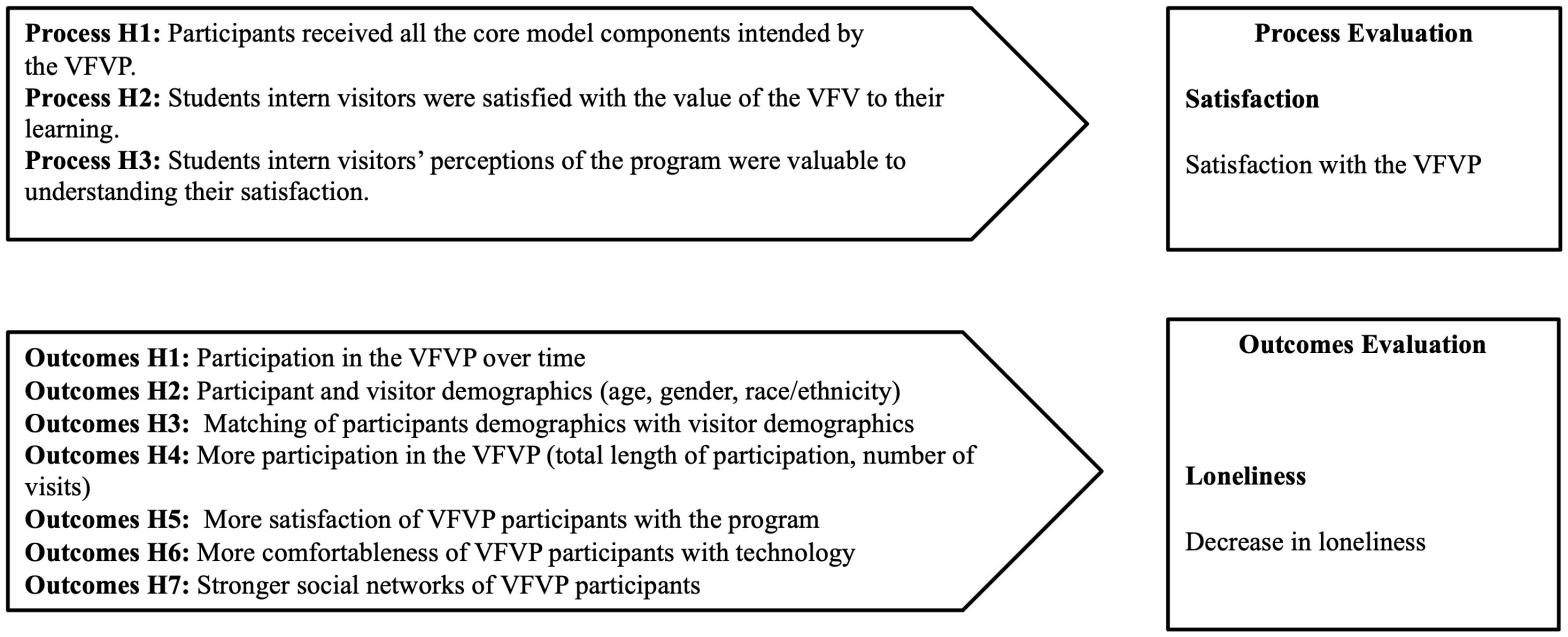
Hypothetical model for study.

The main quantitative research question guiding the outcomes evaluation study was: (1) Would participation in the VFVP be associated with lower levels of loneliness for an older adult? Specifically, the following hypotheses were tested: Outcomes H1: Participation in the VFVP will be associated with reduced loneliness over time; Outcomes H2: Participant and visitor demographics will have an association with reduced loneliness; Outcomes H3: Matching of participants demographics with visitor demographics will have an association with reduced loneliness; Outcomes H4: More participation in the VFVP (total length of participation, number of visits) will be associated with reduced loneliness; Outcomes H5: More satisfaction of VFVP participants with the program will be associated with reduced loneliness; Outcomes H6: More comfortableness of VFVP participants with technology will be associated with reduced loneliness; Outcomes H7: Stronger social networks of VFVP participants will be associated with reduced loneliness. The hypothetical model that drove this study is illustrated in Figure 1.

### Participants and visitors

Our VFVP program recruited a convenient sample of older adults or adults with chronic conditions identified as being socially isolated and lonely. Participants were referred through primary care or community partners. Inclusion criteria was as follows: (1) older adults or adults with chronic health and/or mental health conditions who self-identified as socially isolated and lonely; (2) older adults or adults with chronic health and/or mental health conditions identified by health care team as socially isolated and lonely; (3) older adults who showed an interest to use technology as part of visits with volunteers; and (4) older adults who were available to participate for at least 3 months in weekly virtual visits with a volunteer. Exclusion criteria was as follows: (1) persons with severe cognitive impairment; (2) persons with severe psychiatric conditions with active psychosis; and (3) persons who are under the age of 40. No compensation was provided for participation in the VFVP.

Informed consent for each participant was obtained during the intake process. The informed consent process included a research team member providing a complete description of the program and its purpose, eligibility criteria, explaining that the program is voluntary, risk and benefits of the program, confidentiality, engagement and withdrawal procedures.

Fifty participants agreed to participate in the VFVP between December 2021 and March 2024. Of those 50, 11 completed the intake and baseline assessment, but never engaged with the program. Fourteen completed the intake and baseline assessment and engaged with a few visits with a visitor but dropped out of the program before the second assessment. Twenty-five participants remained for this evaluation study who all had at least two assessments completed.

The twenty-four visitors who visited these 25 participants were all social work student interns completing internship placements at the Institute. Collectively these 24 visitors visited the 25 participants 478 times. Eleven visitors only visited one participant, with the rest visiting between 2 and 4 participants.

### Data collection

For the quantitative outcomes evaluation VFVP participants completed a baseline assessment on loneliness, strength of social networks, and comfortable use of technology, followed by follow-up assessments after the first month and then every 3 months of program participation. For both the quantitative outcomes evaluation as well as the qualitative process evaluation, visitors completed a service record within 48 h after each visit noting the topics discussed, and providing a behaviorally specific, narrative description of the visit. The service record also included a safety check of any disconcerting issues that required immediate action by their supervisor, such as noticeable changes in the appearance of their participant, home environment, ability to talk, affect, confusion or complaints about pain, difficulty breathing or illness. The visitor completed an evaluation survey addressing their perceptions of the VFVP at the conclusion of their commitment.

### Measures

The qualitative process measures of the study included (1) the topic discussed section using a 31-item list, (2) the narrative story of the visit and (3) the visitor evaluation at the conclusion of their commitment. Collectively, these measures acted as fidelity checks for the program objectives.

The quantitative outcome measure for this study was loneliness. Loneliness was operationalized as the subjective feelings of loneliness as measured by the 3-item UCLA Loneliness Scale. The UCLA Loneliness Scale asked the participants to rate on a scale from 1 (hardly ever) to 3 (often) how often did they feel that they lack companionship, how often do they feel left out, and how often do they feel isolated from others. The score was the sum across all items, ranging between 3 and 9. The three item version has shown to be a reliable measure in terms of internal consistency (coefficient alpha = 0.72), convergent and construct validity (46). A total score of 6 or greater signals an individual who is likely to be dealing with loneliness on a regular basis (47). The scale is a robust and reliable tool for measuring loneliness and evaluating the impact of interventions aimed at reducing loneliness and has been used in multiple studies (48). For this study we modified the response options for the UCLA Loneliness Scale asking respondents to rate their responses on a scale from 1 (never) to 4 (always) due to this study being part of a larger study. To effectively use the recommended cutting scores for descriptive purposes, we transformed the individual item scores from 4 point to 3 points. For analysis purposes the 4-point responses were used. Predictor measures were participation in program as measured by number of visits and total minutes visited per participant over time, age, gender and race/ethnicity of participants and visitors, differences between participant and visitor in terms of age, gender and race/ethnicity, participant satisfaction with the visits over time, comfortableness of participant technology use over time, and the strength of social networks over time. All visit encounters and total minutes per visit were recorded in the service record. Age, gender, and race/ethnicity of participants were retrieved from their enrollment documents and for visitors from the internship documents. The visitor age, gender and race/ethnicity were weighted in terms of the proportion of overall visits the visitor had with the specific participant. Participant satisfaction with the program was measured by two questions, namely whether they were satisfied with the visits and the degree to which they would recommend the VFVP to a friend on a scale from 1 (not at all) to 5 (a great deal). Comfortableness of participant technology use during visits was measured on a scale from 1 (not at all) to 5 (a great deal).

The strength of social networks was measured by the abbreviated 6-item Lubben Social Network Scale (LSNS-6) with its two subscales (family and friends). The scale asks 3 questions each about friends and family, specifically how many friends/family did they feel close to such that they could call on them for help, how many friends/family did they feel at ease with that they could talk about private matters, and how many friends/family did they see or hear from at least once a month. The item responses included 0 = none, 1 = one, 2 = two, 3 = three or four, 4 = five thru eight, and 5 = nine or more. The score was the sum across all items, ranging between 0 and 30. A clinical cutpoint score of less than 12 on the LSNS-6 indicated that, on average, the respondents had fewer than two people to perform the particular social networking functions assessed by the LSNS-6. The scale demonstrated high levels of internal consistency, stable factor structures, and high correlations with criterion variables (49). The scale has been widely used to measure an individual’s social networks and the support derived from it, particularly focusing on older adults and has been used to evaluate the effectiveness of social networking programs (50).

### Analysis

For the process evaluation, the qualitative Sort and Sift, Think and Shift data analysis approach was used to analyze the narrative stories of the visits, as well as to provide a narrative understanding of the nuances of the discussed topic during the visits. This approach is an iterative process where our team dived into the narrative stories to understand its content, dimensions, and properties. We then stepped back to assess what we have learned and to determine next steps. We moved from establishing an understanding of what is in the data (“diving in”) to exploring our relationship to the data (“stepping back”). This process was repeated throughout the analysis phase until we arrived at an evidence-based meeting point that is our hybrid story of data content and our own knowledge of the program (51).

The quantitative outcomes study utilized hierarchical linear modeling to test a longitudinal cross-classified growth model with between 2 and 5 measurement occasions (52). It has been used in similar studies to evaluate the influence of social participation on loneliness (53) and to test the mediating effect of social contact on the relationship between internet use and loneliness (54). Hierarchical linear modeling assumes there is a hierarchical structure in the data set and that units of observation fall into groups or clusters. We identified three clusters for this study: level 1 refers to the measurement occasions for each participant, level 2 refers to the older adult participants, and level 3 refers to the visitors visiting the participants. The model is not purely hierarchical in nature with each lower-level unit belonging to a single higher-level unit. Each participant received visits from between 1 and 3 visitors and each visitor visited between 1 and 4 participants, resulting in visitors (level 3) belonging to more than one participant (level 2), and participants belonging to more than one visitor. The model can therefore be classified as a two-way cross-classified model. In these models, the lower-level units do not belong to only one higher level unit. Instead, the lower-level units are nested within multiple higher-level units. Thus, the participants were nested within multiple visitors. It is important to note that in these models, data will vary dependent on the degree to which the lower level units belong to the higher-level units (55). The impact of each visitor on each participant are weighted in this model according to the proportion of the total visits a visitor completed with a participant.

Hierarchical linear modeling was the preferred analysis for this dataset, due to the imbalanced design where the data structure was not uniform across all time periods for all participants. Level 1 measurement occasions varied between a total of 25 participants with up to two measurement occasions, with the rest of the participants with more measurement occasions. The final 5th measurement occasion only had 5 participants. This was a function of some participants who varied in the duration of their participation, and also some inconsistencies in the data collection periods over time due to oversight challenges. The modeling techniques used for this study are specifically equipped to handle such complexities without compromising the integrity of statistical inference (56).

Hierarchical linear modeling allowed for the identification of patterns within and between participants as well as for testing potential interactions between predictors and time (57, 58). Model fit was accomplished with Bayesian modeling using Markov Chain Monte Carlo estimation (67, 70), with the software package MLwiN, version 3.05 (59). All the continuous variables were centered on the grand mean. Centering was done to control for potentially troublesome correlations among random components (60, 61). The model was allowed to vary on the intercept (level 2). The distribution of each variable, including outliers, was inspected and corrected as needed to prevent any violation of functional form in the predictor variables.

The hypotheses were tested in three steps with a focus on understanding changes over time in loneliness and social isolation and potential predictors that could be associated with these changes: (1) fitting the unconditional growth model depicting loneliness over time across individuals (Model A); (2) fitting the demographics of both participants and visitors (Model B), (3) fitting the predictors to explain the outcome variable for individuals (Model C); and (3) fitting the interaction effects of time with the predictors to explain the change in the outcome variable (Model D). In the interest of parsimony, predictor variables that did not contribute to the model fit were excluded from the final models (Models C and D) (52, 62).

Previous studies on FVP interventions for older adults suggest that a small to medium effect size could be expected. Due to the small sample size, *a priori* power analysis suggested that the detection of only a large effect size would be possible for the ideal power of 0.80. To adjust for this problem, we set the alpha level at 0.10 to enable at least a power of 0.80 for a medium effect size.

## Results

### Demographics of participants and visitors

Table 1 provides a summary of the participants in terms of their demographics, their evaluation of the visits, and their loneliness and social isolation scores at each measurement occasion. From the table it is clear that the participants were mostly female and White Non-Hispanic with a mean age of 71 years. On average participants were very satisfied (4) or extremely satisfied (5) with the visits. Also, participants indicated that they would recommend the VFVP to others a lot (4) or a great deal (5). Participants had a moderate amount (3) to a lot (4) of comfort with using technology during the visits. The social network scale showed a baseline score of 8.3 (4.7) indicating weak social networks. Twenty-one participants scored lower than the clinical cutpoint on social networks at baseline. The scores slightly improved over time. Loneliness showed a baseline score of 6.5 (1.5), indicating high levels of loneliness. Twenty two participants scored higher than the clinical cutpoint on loneliness at baseline. The scores slightly decreased over time.

**Table 1:** Visit data per participant.

Older adult	# visits	Total minutes	# MO**	Total Vs**	Visitor 1 ID	Weight V1	Visitor 2 ID	Weight V2	Visitor 3 ID	Weight V3
1 (f,w)*	2	118	2	2	6 (f,o)	0.50	16 (f,w)	0.50	0	0
2 (f,w)	9	282	2	1	8 (f,w)	1.00	0	0	0	0
3 (f,o)	4	120	2	2	5 (f,w)	0.50	23 (f,w)	0.50	0	0
4 (f,w)	20	977	3	1	23 (f, w)	1.00	0	0	0	0
5 (m,w)	22	940	4	3	25 (f,w)	0.68	10 (f,w)	0.27	15 (f,o)	0.05
6 (m,w)	23	1,310	4	2	18 (m,w)	0.91	21 (m,w)	0.09	0	0
7 (m,w)	4	163	2	1	4 (f,w)	1.00	0	0	0	0
8 (f,w)	5	422	2	1	4 (f,w)	1.00	0	0	0	0
9 (f,o)	17	1,370	2	1	18 (m,w)	1.00	0	0	0	0
10 (f,w)	63	3,700	5	3	3 (f,w)	0.69	8 (f,w)	0.30	25 (f,w)	0.01
11 (f,o)	30	2023	5	3	10 (f,w)	0.44	17 (f,w)	0.33	2 (f,w)	0.23
12 (f,w)	7	225	2	1	22 (f,w)	1.00	0	0	0	0
13 (f,o)	3	121	2	1	20 (f,w)	1.00	0	0	0	0
14 (f,w)	28	1,468	2	2	9 (f,w)	0.57	23 (f,w)	0.43	0	0
15 (f,w)	21	1,077	2	1	3 (f,w)	1.00	0	0	0	0
16 (m,w)	42	1769	5	3	9 (f,w)	0.32	19 (f,o)	0.24	21 (m,w)	0.22
17 (m,o)	7	347	2	1	11 (f,o)	1.00	0	0	0	0
18 (f,w)	17	650	2	1	11 (f,o)	1.00	0	0	0	0
19 (m,w)	3	301	2	1	11 (f,o)	1.00	0	0	0	0
20 (f,w)	18	1,297	3	3	2 (f,w)	0.46	23 (f,w)	0.46	24 (f,w)	0.08
21 (f,w)	10	446	3	2	12 (f,w)	0.60	4 (f,w)	0.40	0	0.00
22 (f,o)	22	1,168	4	3	7 (f, o)	0.42	14 (f,w)	0.42	1 (f,o)	0.16
23 (m,w)	45	1929	5	3	19 (f,o)	0.58	2 (f,w)	0.33	5 (f,w)	0.09
24 (m,w)	32	1827	3	2	19 (f,o)	0.56	2 (f,w)	0.44	0	0
25 (f,w)	24	854	5	3	16 (f,w)	0.54	10 (f,w)	0.27	14 (f,w)	0.19

*f, female; m, male; w, white; o, other. **MO, measurement occasions; V, visitor.

Table 2 provides a summary of the visitors in terms of their demographics, their evaluation of the participants directly after the visits as well as their own evaluation of the visits. From the table it is clear that the visitors were mostly female and White Non-Hispanic – similar to the participants. The mean age of the visitors were 40 years younger than the participants. The visitors felt that the participants benefitted a fair amount (4) to a great deal (5) from the visits. They felt the same about their own benefit they got from the visits. On average the visitors showed a fair amount (4) to a great deal (5) of satisfaction with the outcome of the visit. At the beginning of the program, the skills of the visitors were in the mid-range with them rating their own roles/responsibility skills slightly higher than their communication skills.

**Table 2:** Participant demographics, visit evaluations, loneliness and social isolation.

Demographics					
	*f*		*f*		Mean (SD)
Gender		Race and Ethnicity		Age	71.7 (8.2)
Female	17	White Non-Hispanic	19		
Male	8	Other	6		

Evaluation of VFVP (Mean (SD))					
	Time 1 (*n* = 25)	Time 2 (*n* = 25)	Time 3 (*n* = 13)	Time 4 (*n* = 8)	Time 5 (*n* = 5)
Satisfaction with visits (1–5)	4.2 (0.8)	4.5 (0.6)	4.8 (0.4)	4.5 (051)	4.4 (0.5)
Will recommend VFVP (1–5)	4.3 (0.8)	4.4 (0.7)	4.5 (0.7)	4.8 (0.5)	4.8 (0.4)
Comfort with technology and social networks [Mean (SD)]					
Comfort with technology (1–5)	3.5 (1.3)	3.9 (1.1)	3.9 (1.2)	3.6 (1.3)	3.8 (1.1)
Social Networks (0–30)	8.3 (4.7)	10.2 (4.7)	10.8 (6.7)	13.0 (7.7)	10.8 (5.2)
Social Networks below clinical cutpoint of 12 (f)	21	17	6	4	3
Loneliness [Mean (SD)]					
Loneliness (3–9)	6.8 (1.5)	6.4 (0.9)	6.6 (1.2)	6.5 (0.8)	6.2 (0.8)
Loneliness above clinical cutpoint of 6 (*f*)	22	25	12	8	4

### Intervention

Twenty-four visitors completed 478 visits with 25 older adult participants. The number of visits per participant ranged between 2 and 63 visits for a mean of 19.1 visits (15.1). Total overall minutes visited per participants ranged between 118 (±2 h) and 3,700 min (±62 h) for a mean of 996.2 min (SD = 839.0) (±17 h). The total number of measurement occasions ranged between 2 and 5 for a total of 76 measurement occasions. Nine outlier measurement occasions were excluded from analysis. Twelve participants had more than 2 measurements occasions.

Table 3 shows the gender and race/ethnicity of all the participants and their visitors, the total number of visits per participant, the total minutes visited per participant, the number of measurement occasions, the total number of visitors who visited the older adult participants, as well as the proportion of visits each visitor completed with an older adult participant while they were in the VFVP. Eleven older adults only had 1 visitor.

**Table 3:** Visitor demographics, visit evaluations, and skill.

Demographics					
	**f**		f		Mean (SD)
Gender		Race and Ethnicity		Age	31.7 (10.2)
Female	22	White non-hispanic	18		
Male	2	Other	6		

Evaluation of participant after visit (Mean (SD))					
	Time 1 (*n* = 92)	Time 2 (*n* = 201)	Time 3 (*n* = 98)	Time 4 (*n* = 40)	Time 5 (*n* = 47)
Benefitted from visit (1–5)	4.3 (0.8)	4.6 (0.7)	4.5 (0.9)	4.3 (0.8)	4.4 (0.9)
Visitor evaluation after visit [Mean (SD)]					
Benefitted from visit (1–5)	4.1 (0.1)	4.5 (0.8)	4.6 (0.8)	4.1 (0.9)	4.4 (0.9)
Satisfied with outcome (1–5)	4.4 (0.8)	4.6 (0.8)	4.6 (0.9)	4.3 (0.8)	4.4 (0.9)

### Process evaluation results–narrative analysis

Table 4 provided the most frequently discussed topics during the visits which included health and well-being concerns, personal histories, recreational activities, validation of feelings, social networks and practical support and advocacy. Example excerpts from the narrative descriptions are also provided in the table to give greater context to the topics frequently discussed.

**Table 4:** Main visit topic categories and corresponding visitor quotes during the visits.

Health and well-being concerns
“She has been experiencing pain in her left leg from a stroke she suffered a year ago.” “She worries about the long-term effects of her medication.” “She told me during our conversation that they were trying to place her in a nursing home against her will.” “She feels that her health issues are not taken seriously by her healthcare providers.” “He spoke about the challenges of navigating the healthcare system.” “She will need surgery to her rotator cuff, this has been bothering her and how she’ll be able to maintain her level of care for herself after surgery.” “He was very thankful the fall had not resulted in any injuries that would have put him in care.”
Personal histories
“Memories from her childhood were shared, including stories of gardening experiences.” “He expressed his father was so good to him and he wished he could have lived with him, instead of his mother who was never home.” “She shared some childhood trauma and we discussed how it shaped who she became as an adult.” “We talked about her former career and she shared stories from her working days.” “We also talked about past travels and movies and hot air balloons and planes.” “We also discussed her childhood and how it was growing and how she decided to raise her children differently.”
Recreational activities
“He will be zooming with his daughters soon. We spoke about his grandchildren and that he even has great-grandchildren.” “We talked about life with tinnitus, Celtics music, and different sound frequency healing.” “He went on at length about his career as a photographer and how he would like to get back into it as a hobby and possible source of income.” “We then finished our conversation talking about music and his previous involvement in his community band.” “We ended the phone call with her talking about one of her favorite TV shows, “Married At First Sight.” She told me about some of the contestants and how the show works.” “She and her husband went out to eat, and they stayed outside while eating.” “We spoke about sports and our German heritage.”
Validate feelings and social networks
“She has been nervous about her move and spoke mostly about that. She said she was very thankful for having me available to talk to.” “He does not have a lot of social support and only 1 living family member, his brother.” “Susan expressed sadness because she had an aunt and uncle that are currently very sick and she was told they would probably pass away this week. I provided empathetic responding and asked her about her favorite memories with them.” “She confided in me about some family difficulties she was having.” “We spoke about his concerns and fears regarding his upcoming cataract surgery.” “We talked about the trauma from losing her friend recently to death.”
Practical support and advocacy
“We discussed her memory lapses and ideas for self-care practices such as her journaling and meditations.” “I recommended he call the clinic back and inquire more about the missed appointment.” “I told her she needs to contact her PCP for these concerns that she has.” “I made sure that she has been drinking more water because she promised me that she would.” “I told her that it is ok to change her PCP (she feels guilty to do so), and that her health is priority and that she should not feel bad for putting herself first.” “She talked about how she misses being able to rent movies at local video store due to it closing down. I recommended renting books/movies at local library.” “She had previously asked about applying for SNAP so I shared a number for intake assessment she could call.”

#### Thematic analysis of narrative review description

The analysis of the narrative description of the visits provided insight to the implementation fidelity of the core components of the VFVP, highlighting the mechanisms by which the visitors reduced the social isolation and loneliness of the older adults. The visit themes noted the program gave the older adults an outlet to share a detailed enumeration of their day-to-day activities, encouraged the student interns to dilute the negative thought patterns by infusing positive affect into the lives of the older adults, provided older adults access to immediate emotional support and resources that was missing from their lives and allowed for deep, mutual exchange of core values between the older adults and student visitor which offered intimacy and trust. The names in the following quotes used to highlight the aforementioned have been changed to protect the anonymity of the older adult participant.

Some student interns wrote about the older adult visitors sharing highlights of their activities, celebrations, and milestones of the past week. One student intern consistently shared in their weekly service records that his older adult participant typically delineated the hassles and fun filled aspects of his week. The student visitor wrote in one entry:

*“…Started conversation with Adam explaining about how he lost his phone last week. He went through the story about all the places he went to look for it only to end up finding it under a table. We talked about his new coffee maker and how he had his mother and brother over for coffee last weekend. He expressed excitement over seeing family this coming weekend to watch the game. We ended our conversation by talking about Adam’s favorite show, Star Trek.”*

Other student interns highlighted in their narrative whether they were successful in their desired outcome of increasing the positive affect of the older adult by uplifting their spirits or providing laughter for the older adult during their visits.

*“Bonnie has been struggling more since she lost her therapist. She talked about wishing her family would spend more time with her and how she has struggled more with everything since her stroke. By the end of the visit, she sounded in better spirits and said she was excited about our next video visit.”*

*“Troy claimed; “I am too old.” I follow with; “Are you being pessimistic?” Troy then states, “I am double your age!” I reply “And double wisdom!” This prompted a big laugh from Troy. I try to help reframe things he sees as negatives as strengths.”*

*“Dolores is really concerned about the health issues her daughter is having. She mentioned how she likes to maintain her yard by pulling and trimming weeds. She has a new nurse coming to check on her next week. She told me how happy she is when I call her and how it makes her feel better.”*

Other student interns shared in their narrative descriptions that their older adult participants struggled with their own and family members’ health challenges, both noting the volume of challenges or losses, and the inability to share their despair or deconstruct their emotions surrounding the health challenges with their family members. Excerpts from three student interns demonstrate similar experiences of grief for the older adults originating from different health circumstances:

*“She explained her frustrations trying to get back to the dermatologist to check in about her cancer. She had recent dental work but thankfully they were able to fix it. Her daughter is dealing with a health issue, and she will not disclose what it is. This has been very upsetting for her; she began to cry during our session. She mentioned again how helpful talking about these things with me is.”*

*“Mary has been struggling with how to handle the news that she has dementia. She is going through stages of anger and wants more information. We talked about how her family does not want to talk about her feelings and that she is scared about what having dementia will mean. She talked about having to take care of her mom when she got sick and that she does not believe that any of her family can take care of her.”*

*“Mae shared she had 3 brothers and 2 sisters and that they had all passed away. Then she shared that her mother passed away last. She said she is the only one left. Her emotions seems as if she accepted the losses but misses them.”*

Some student intern visitors shared in their narrative the flexibility they afforded their older adult participant for virtual visits highlighting their desire to be available in times of urgent need. The student interns shared their support, use of non-judgmental approaches and offerings of practical tips to address health concerns. Several interns’ narratives are provided below.

“*Sue Ellen had texted me to see if I was available for a meeting today, to which I replied that I could meet with her. We met* via *Zoom, and she was a bit distressed, as she and her husband had a bit of an argument beforehand. We discussed everything, and I validated Sue Ellen’s feelings, used a strengths-based perspective, and encouraged her to utilize self-care and coping strategies. By the end of the session, she seemed to be in better spirits.”*

*“She has been having some side effects from her medication and I suspect it is due to her not hydrating (muscle cramping). She admitted to me that she rarely drinks water and I told her how important it was to do so. I mentioned other hydrating options as well (after she speaks to her doctor). I told her she can call me anytime if she needs to speak to someone, even if it is more than once per week.”*

*“I talked to him about his rights as a patient and that it is important that he advocate for himself and ask for help, if that is not working. He asked about contacting me if something comes up to change his weekly appointment or if there is another issue. I told him to call my cell phone and, if I do not pick up, leave me a message and I will get back to him.”*

While other student interns wrote about their engagement in deep discussions of private matters including the mutual exchange of intimate aspects of both the visitor and participants’ lives, such as family and faith. Below represents examples of the aforementioned.

*“She shared her experience since her stroke, and I shared my experience with my husband’s brain injury. We laughed and shared many stories; some were painful memories; however, they seemed to inspire us both. She opened up about her struggles and triumphs.”*

*“We spoke at length about what God truly means, what it encompasses, how it relates to the vast universe and more. We spoke about connections throughout our lives and how knowing oneself is as important as knowing others. She described the process she is currently going through about finding purpose since her stroke.”*

*“We talked about our Christmas traditions and her Thanksgiving. She gave me great advice about how to connect with my grandmother who has dementia. She also told me about changes she’s noticed from her childhood until now.”*

*“The conversation touched upon “his envy” of my upbringing that I had shared related it to his perception of his own challenging childhood. We acknowledged life’s inherent unfairness.”*

Analysis of the themes inherent in the narrative stories suggest that the implementation of the core aspects of the VFVP was achieved, confirming implementation fidelity. Themes revealed that the program was successful in creating new and meaningful relationships and provided informal support as well as mediated social networks (Process H1).

#### Thematic analysis of student visitor narrative description of perceived value and benefits

All of the student interns commented on the perceived value and positive impact of the program, emphasizing the program’s role in improving the lives of the older adult participants. Some of the interns continued their visits with the older adult participant beyond the conclusion of their 2-semester commitment. A few student intern visitors also planned face to face, in person visits to their older adult participants after their commitment ended. One student intern visitor commented, “Great program. I do not want to graduate and leave my participants.” Another student visitor reported, “My VFVP person and I have gained a wonderful friendship through this program. She has stated several times how beneficial this program has been. I also receive great benefits from visiting weekly with her.”

Student intern volunteers shared insights into how their involvement has facilitated personal growth and professional skills development, particularly in areas relevant to social work like empathy, active listening, and motivational interviewing. A few reflections are noted below.

*“I have found that throughout my time working here that I have been able to develop different skills that I find necessary to be the most competent social worker possible. I gained skill in developing rapport with my one participant. I would really love to gain more so I could talk to others and really work on my skills as a social worker when it comes to empathy, active listening and motivational interviewing.*

*“I spoke with participants on an individual level in virtual friendly visitor program sessions. This was my first time having so much freedom to practice social work skills.”*

Intern visitors often spoke of the admiration and respect for the older adult and at times a reduction of negative stereotypes and attitudes toward aging. Excerpts from two social work intern visitors are offered below.

*“I always feel that after our sessions she has somehow benefitted from our conversations. And I benefit from them too, I have grown close to Tracy. I like her personality and her determination to overcome her obstacles. She has amazing self-care skills, she loves to read books, and watch cooking shows or dramas. She is a caregiver and much of her time is taken by her weekly tasks and duties but she always keeps a positive attitude.”*

*“I also have gained a better insight on the older adult population and how they really are just people who are still living and not just wasting away.”*

The social work interns also discussed various challenges such as the older adult participant’s adjusting to the program’s demands following medical events (e.g., strokes, hospital admissions, diabetes complications), technical issues with equipment and software, and initial hurdles of getting the visits established and sustaining the visits due to various scheduling changes in the older adult’s life including medical visits.

The above narrative highlights the fact that the social work interns expressed satisfaction with the VFVP, noting its significant impact on their learning and personal development. They highlighted growth in key social work skills such as empathy and active listening, and many interns expressed a strong connection with their older adult participants, with some continuing their visits beyond the program’s formal duration (Process H2). The social work interns’ positive perceptions of the program were crucial in understanding their overall satisfaction, as reflected in the emotional bonds they developed with participants and acknowledging previously held stereotypes toward aging, all contributing to a meaningful and fulfilling learning experience (Process H3).

### Outcomes evaluation results

The final data on the outcomes model is shown in Table 5. Model A shows a significant decrease in loneliness over time for participants. The final Model D was able to explain most of the decrease in loneliness over time, resulting in time no longer being significant in the final model. Significant predictors that could explain loneliness differences for participants as a function of the visitors they had (level 3) were age and race/ethnicity. Younger visitors and White visitors in general visited with lonelier participants. Significant predictors that could explain loneliness differences between participants (level 2) were also age, and race/ethnicity such that younger participants and those of other race/ethnicity than white was the loneliest. In addition, people who spent the most time in the program were also the loneliest. Significant predictors on the measurement occasion level (level 1), were if participants were ready to recommend the program to others, if participants were satisfied with the visits, if participants were comfortable with the technology, and if the participants felt they had strong social networks. These variables were all measured every time the loneliness outcome measure was completed. The more people were willing to recommend the program, the more loneliness decreased. The same was true for networks, the more social networks improved, the more loneliness decreased. However, for satisfaction with visits and comfortableness with technology, the association was in the opposite direction, meaning that as satisfaction with visits increased, loneliness also increased. The same was true for comfortableness with technology, the more they become comfortable with technology, the more loneliness increased.

**Table 5:** Loneliness.

	Model A	Model B	Model C	Model D
Fixed effects				
Constant_0ijk_	9.27 (0.37)***	9.74 (1.45)***	10.33 (1.29)***	9.87 (1.01)***
Time_ijk_	−0.26 (0.13)*	−0.21 (0.14)~	−0.21 (0.13)*	0.20 (0.24)
Participant age_jk_		−0.10 (0.04)**	−0.09 (0.04)**	−0.11 (0.03)***
Participant female_jk_		0.01 (0.73)	−0.18 (0.60)	0.01 (0.41)
Participant white_jk_		0.99 (0.73)~	1.03 (0.56)*	1.71 (0.50)***
Visitor weighted age_jk_		0.02 (0.05)	−0.05 (0.05)	−0.08 (0.03)*
Visitor weighted female_k_		0.25 (1.26)	−0.20 (1.12)	−0.57 (0.81)
Visitor weighted white_k_		−2.15 (0.92)**	−2.18 (0.79)***	−1.79 (0.53)***
Total visit time (minutes)_jk_			0.07 (0.03)**	0.07 (0.02)***
Participant satisfaction_ijk_			0.89 (0.28)***	1.18 (0.32)***
Participant recommend program_ijk_			−1.07 (0.28)***	−1.21 (0.26)***
Participant comfort with technology_ijk_			0.36 (0.15)**	0.47 (0.14)***
Social networks_ijk_			−0.04 (0.04)	−0.06 (0.04)*
Time _ijk_ × Participant age_ijk_				0.03 (0.02)~
Time _ijk_ × Participant white_ijk_				−0.54 (0.27)*
Time_ijk_ × Participant satisfaction_ijk_				−0.43 (0.21)*
Total visit time _jk_ × Social networks_ijk_				−0.01 (0.00)*
Total social networks_ijk_ × Comfort with technology_ijk_				−0.04 (0.02)*
Random Parameters				
Level: visitor				
Var (Constant)_0k_	0.40 (0.78)	1.29 (1.79)	0.58 (1.12)	0.13 (0.32)
Level: participant				
Var (Constant)_0jk_	1.76 (0.78)	0.94 (0.86)	0.58 (0.51)	0.11 (0.19)
Level: measurement occasion				
Var (Constant)_0ijk_	1.57 (0.33)	1.61 (0.35)	1.27 (0.30)	1.17 (0.24)
Units: Visitor	24.00			
Units: Participant	25.00			
Units: Measurement Occasion	76.00			
Estimation:	MCMC			
DIC:	269.05	271.58	255.36	248.56
pD:	20.93	21.35	23.63	22.01
Burnin:	500			
Chain Length:	50,000			
Thinning:	1			

Standard errors are in parentheses. ∼*p* ≤ 0.10; ∗*p* ≤ 0.05; ∗∗*p* ≤ 0.01; ∗∗∗*p* ≤ 0.001. DIC, diagnostic information criterion; pD, estimated degrees of freedom.

**Figure 2:**
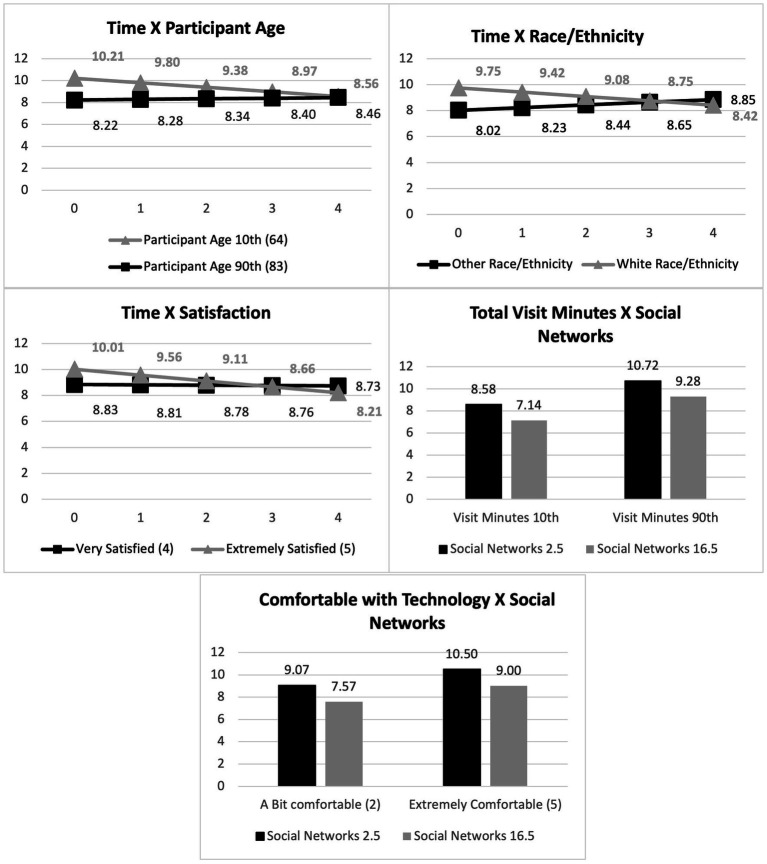
Significant interaction effects.

A few interactions showed interesting results and are illustrated in Figure 2. The interaction between time and participant age, showed that the younger participants declined the most in their loneliness with older participants slightly increasing on their loneliness. The interaction between time and race/ethnicity showed that other race/ethnicity participants increased on their loneliness, while white participants decreased in their loneliness. Interaction between time and satisfaction with visits showed that participants who were very satisfied (10th percentile) showed no change in loneliness compared to those who were extremely satisfied (90th percentile) who showed a significant decline in loneliness. The interaction between social networks (level 1) and total visit minutes (level 2) showed that those with the least social networks over time were also those who stayed in the program the longest and were the loneliest while in the program. The least lonely participants were those who stayed the shortest in the program with the most social networks. Another interesting interaction was between social networks and comfort with technology. The loneliest participants had the least social networks and were extremely comfortable with technology. The least lonely participants had the most social networks and were only slightly comfortable with technology.

Total visits completed, as well as the skills of the visitors did not show any significant effect and were excluded from the analysis. The final model showed a significantly improved model fit (DIC reduced from 271.59 to 248.56). Based on the variance partition coefficients, the original variance (Model A) was mainly on level 2 (participant) at 47%, followed by level 1 (measurement occasions) (42%), with the least variance on level 3 (visitor) (11%). After the predictors were added, there was an overall decrease in remaining unexplained variance, with the most variance explained on level 2. Other factors related to the visitors and measurement occasions not tested in this study may explain the remaining unexplained variance on levels 1 and 3.

## Discussion

The results indicate that participation in the VFVP is associated with significantly reduced loneliness over time, supporting the first hypothesis (Outcomes H1). This aligns with previous research that highlights the benefits of structured social programs for older adults in alleviating feelings of isolation and loneliness (36). The longitudinal design of the study strengthens the validity of this finding by capturing changes in loneliness across multiple time points. Interestingly, the study found that the total length of participation and the number of visits (Outcomes H4) were not significantly associated with loneliness reduction. This suggests that simply increasing the quantity of interactions over time by a visitor is insufficient to impact loneliness significantly. The quality and content of interactions by the visitor may play a more critical role, as indicated by the significant predictors identified in the study. Additionally, having multiple visitors during a specific time period instead of one visitor or having group visits may be warranted to decrease loneliness suggesting dosage may have not been sufficient to combat the loneliness (32, 63).

This study showed that younger participants and White participants benefitted the most by participating in the program as their loneliness declined the most as compared to older participants and other race/ethnicity participants who increased in their loneliness (Outcomes H2). This speaks to the fact that virtual visitor programs may benefit younger participants more due to their comfortableness with virtual interactions, finding them more engaging and convenient than older adults who may still rather prefer face to face interactions. White participants may have benefited more from the virtual visits due to mostly being visited by White visitors. Even though Hypothesis 3 (Outcomes H3) showed that similarities between visitors and participants based on race/ethnicity did not result in reduced loneliness of participants, this finding could have been attributed to power concerns as there were only 6 participants and 6 visitors that were not White Non-Hispanic. Other studies have shown that matching visitors and participants on demographics may indeed have benefits for the success of the program (12).

Participants being extremely satisfied with the visits and their willingness to recommend the program were significantly associated with reduced loneliness (Outcomes H5). This underscores the importance of participant engagement and their perceived value of the program, suggesting that programs like the VFVP should prioritize participant satisfaction to enhance their effectiveness. In fact, data has shown that the overall success of these programs is more closely linked to the quality and meaningfulness of the interaction, mutual respect, and shared activities between the volunteer and the older adult, leading to more satisfaction with the program (12).

The finding that greater comfort with technology was associated with increased loneliness (Outcomes H6) was unexpected. This may indicate that while technology facilitates social interactions, overreliance or increased comfort with technology may lead to more screen time and less face-to-face interaction, potentially exacerbating feelings of loneliness (69). This is supported by literature indicating that excessive internet use can lead to social isolation and increased loneliness, especially when the Fear of Missing Out (FoMO) is strong – fears that were very prominent during the COVID-19 lockdown and thereafter. Studies have suggested that individuals experiencing high levels of FoMO are more likely to engage in problematic social networking site use. FoMO drives individuals to increase their online communication and seek relational closeness through social media to mitigate their fears of missing out on social interactions. This can lead to unhealthy, compulsive behaviors online, highlighting the complex relationship between social media use, FoMO and loneliness (64, 65). Therefore, while technology can be a powerful tool for maintaining social connections, it is crucial to use it mindfully and engage in meaningful interactions to avoid the negative consequences associated with excessive and passive use (66, 68). Additionally, having an occasional in-person visit, if possible, to complement the frequent virtual visits may also reduce the loneliness, especially for those who are older and less confident in using technology.

The improvement in social networks was associated with reduced loneliness (Outcomes H7), confirming the critical role of befriending programs to improve social networks and mitigating loneliness. The interaction effects revealed nuanced relationships between social networks, technology use, and loneliness, suggesting that the interplay between these factors is complex and context dependent.

The study’s findings have several practical implications. For instance, ensuring high levels of participant satisfaction and promoting meaningful engagement are crucial for the success of programs like the VFVP. Additionally, while fostering technological skills among older adults is important, it is equally vital to balance this with opportunities for in-person interactions to prevent increased loneliness. Future research should explore the qualitative aspects of interactions that contribute to loneliness reduction. Moreover, examining the long-term impacts of such programs and their sustainability will provide further insights into their efficacy.

## Limitations and future program directions

While this study provides valuable insights into the value of a friendly visitor program on loneliness among older adults, we had several limitations that should be considered. This study experienced attrition in the program and could only utilize 25 of the 50 conveniently sampled, mostly White female older participants and 24 mostly White female visitors, which limits the generalizability of the findings. A larger sample size with a more balanced race/ethnicity and gender distribution would provide more robust and generalizable results.

Additionally, the participants and visitors were predominantly white, non-Hispanic females. This lack of diversity may limit the applicability of the findings to other demographic groups and individuals from different racial/ethnic backgrounds. Also of note, there was considerable variability in the number of visits and the total minutes spent with the participants. This variability makes it challenging to standardize the intervention and may affect the consistency of the results. In addition, our study might not have accounted for other factors that could influence loneliness, such as physical health and mental health status. An additional concern is the use of self-report measures in which older adults may experience heightened social desirability and recall biases in their responses associated with a service that is both desired and limited in availability. Additional attention will be taken in future studies to be sensitive to these concerns.

Lastly, our program evaluation design was not optimal as assessments on loneliness and social networks were completed outside the context of the visit, resulting in student interns not always completing the assessments with their participants on time or not completing them at all, resulting in the imbalanced design of only 25 participants with at least 2 measurement occasions and only 5 participants with 5 measurement occasions. Future evaluation design protocols will include assessments at each visit to ensure richer data. The assessments will also be expanded to include a basic depression and mood assessment, as well as a measure of emotional, informational and instrumental support provision of the visitor along with the assessment of perceived loneliness and social networks.

While using interns has been an invaluable aspect to the success of our program, it also presents us with a unique problem. Due to the structure of the intern’s academic calendar, the length which the intern volunteers are with us, varies. We have identified that this is a barrier at times, especially when a participant has developed a strong companionship with the retiring intern. Additionally, this can result in participants been assigned a new intern every 6 or 12 months. This may impact a participant’s ability to develop comfortability and rapport with interns, especially if there are multiple transitions. To minimize transitioning for participants, we are working with interns to ensure they can be active volunteers for at least 10 months. As previously mentioned, we have developed a thorough transition process.

This study demonstrates that the VFVP is associated with reduced loneliness among older adults, with participant satisfaction and social networks playing significant roles in this outcome. The complex relationships between technology use, social networks, and loneliness highlight the need for a balanced approach in designing interventions for older adults. The findings contribute to the understanding of how structured social programs can enhance the well-being of older adults and offer directions for future research and practice.

## Data Availability

The raw data supporting the conclusions of this article will be made available by the authors, without undue reservation.
